# Steroid responsive idiopathic calcitriol induced hypercalcemia: a case report and review of the literature

**DOI:** 10.1186/s12882-023-03203-4

**Published:** 2023-06-06

**Authors:** Omar El Fadel, Anshel Kenkare, Jingjing Zhang

**Affiliations:** 1 Sidney Kimmel Medical College, Thomas Jefferson University, Philadelphia, United States; 2 Department of Internal Medicine, Division of Nephrology, Thomas Jefferson University, Philadelphia, United States

**Keywords:** Calcitriol induced hypercalcemia, Idiopathic, Prednisone, Nephrolithiasis

## Abstract

**Background:**

Idiopathic Calcitriol Induced Hypercalcemia is a rare cause of a common condition of hypercalcemia. Hypercalcemia is most commonly the result of hyperparathyroidism and together with hypercalcemia of malignancy accounts for over 95% of cases. Idiopathic Calcitriol Induced Hypercalcemia can mimic hypercalcemia secondary to granulomatous diseases like sarcoidosis, but with apparent absences of both imaging and physical exam findings consistent with the disease. We report here a 51-year-old man who presented with recurrent nephrolithiasis, hypercalcemia, and acute kidney injury.

**Case presentation:**

A 51-year-old man presented with severe back pain and mild hematuria. He had a history of recurrent nephrolithiasis over the course of a 15-year period. On presentation his calcium was elevated at 13.4 mg/dL, creatinine was 3.1 mg/dL (from baseline of 1.2), and his PTH was reduced at 5 pg/mL. CT abdomen and pelvis showed acute nephrolithiasis which was managed medically. Work up for the hypercalcemia included an SPEP which was normal, Vit D,1,25 (OH)2 was elevated at 80.4 pg/mL, CT chest showed no evidence of sarcoidosis. Management with 10 mg prednisone showed marked improvement in the hypercalcemia and he no longer had any symptoms of hypercalcemia.

**Conclusion:**

Idiopathic Calcitriol Induced Hypercalcemia is a rare cause of hypercalcemia. All reported cases benefit from more intensive long-term immunosuppression. This report helps consolidate the diagnosis of Idiopathic Calcitriol Induced Hypercalcemia and encourages researchers to better investigate its underlying pathogenesis.

**Supplementary Information:**

The online version contains supplementary material available at 10.1186/s12882-023-03203-4.

## Background

Symptomatic hypercalcemia with subsequent nephrolithiasis is not an uncommon problem in the clinical arena. The vast majority of patients present in the context of primary hyperparathyroidism or lymphoma [1]. Alternatively, an important category for consideration is granulomatosis disease such as sarcoidosis, tuberculosis, fungal infections, leprosy, and Crohn’s disease [1, 2]. The unifying mechanism, best exemplified by sarcoidosis, is associated with increased extra-renal 1α-hydroxylase activation in macrophages, leading to increased 1,25(OH)_2_D (calcitriol) levels [2, 3]. Less common causes, such as the injection of silicone for cosmetic purposes [4] and paraffin oil in young male bodybuilders, [5] can lead to granulomatosis inflammation and hypercalcemia through a similar mechanism.

Other endocrine disorders could also present with symptomatic hypercalcemia encompass adrenal insufficiency, primary hyperparathyroidism, vitamin D or A intoxication [1]. The differential is broad and the following work-up emphasizes common etiologies, yet idiopathic calcitriol induced hypercalcemia has emerged as a rare diagnosis of exclusion over the years. Only a few case reports [1, 6, 7] have reported cases of idiopathic calcitriol induced hypercalcemia with successful treatment using corticosteroids. Our aim is to add to this literature to reaffirm this possible diagnosis without elevation of angiotensin converting enzyme (ACE). We therefore present the case of a man with recurrent nephrolithiasis, hypercalcemia, and acute kidney injury.

## Case presentation

A 51-year-old man originally from East Asia with past medical history of significant for hypertension was referred to nephrology due to recurrent kidney stones and elevated creatinine. The patient reports having kidney stones in 1989 and 2006 that passed spontaneously. In 2017, the patient was found to have bilateral symptomatic calcium kidney stones requiring lithotripsy. His kidney function has fluctuated (Cr 0.9–3.1 mg/dL) over the preceding two years, with consistent hypercalcemia, hypercalciuria (637 mg/24 hours), and low PTH level.

Seeing this patient for the first time in July 2019, initial workup for possible causes of hypercalcemia included intact PTH (iPTH) suppressed at 5 pg/mL, calcitriol (1,25-dihrdoxyvitamin D ) elevated at 80.4 pg/mL, calcidiol (25-hydroxyvitamin D) low at 15.1 ng/mL, parathyroid hormone-related peptide (PTHrP) normal (< 2.0 pmol/L), serum protein electrophoresis (normal), ACE levels low at 2 U/L, complete blood count (normal), an elevated serum calcium (11.9 mg/dL) and creatinine (1.8 mg/dl), a whole-body bone scan (normal), and urinalysis (normal). Although not necessary, an ultrasound of the neck was also obtained and was found to be normal.

Chest X-ray showed a calcified granuloma. CT scan showed an upper lobe solid nodule concerning for possible primary lung malignancy independent workup through pulmonology were negative for tuberculosis, sarcoidosis, and lung malignancy.

During subsequent visits, he had persistently elevated serum calcium level (11.3 mg/dL) and ionized calcium level (6.0 mg/dL). Given the lack of alternative diagnoses, idiopathic calcitriol induced hypercalcemia was pursued as a primary diagnosis and the patient was started on prednisone 30 mg daily in December 2019.

## Outcome and follow up

Upon follow-up a few weeks later, the patient’s calcium decreased (10.3 mg/dL) and iPTH has increased (13 pg/mL). Urine calcium also decreased from 681 mg to 581 mg and then 386 mg gradually. Prednisone was gradually tapered down to 20 mg daily and then 10 mg daily as we continued to monitor the patient’s labs in 2020. Hypercalcemia continued to improve (9.9 mg/dL) and prednisone down to 5 mg daily in July 2021. Unfortunately, calcium increased to 11.8 mg/dl in 3 months, iPTH was suppressed again, so the prednisone was titrated up to 10 mg daily with calcium controlled around 10 mg/dL. While the patient was taking prednisone 10 mg daily, suddenly developed the kidney stone again in July 2022. The patients’ records were reviewed and it was determined another physician placed the patient on ergocalciferol 50,000 unit weekly for vitamin D deficiency in March 2022 without a full grasp of the patient’s clinical presentation. However, serum calcium was 11.6 mg/dl and calcitriol was 104 pg/ml in July 2022, prompting the nephrology team to discontinue ergocalciferol. Subsequently, calcium dropped down to 10 mg/dL in January 2023. The patient has been on prednisone 10 mg daily for 3 years.

**Fig. 1:**
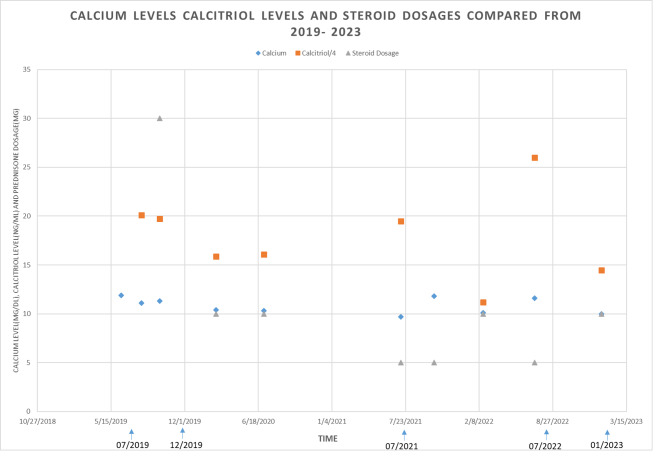
Timeline of calcium level (mg/dl), calcitriol level (ng/dl) and prednisone dose (mg) over time (months). To fit the scale, the calcitriol level was divided by 4

## Discussion and conclusions

The patient described has a long history of symptomatic hypercalcemia and recurrent nephrolithiasis. A broad work-up was pursued eliminating common etiologies of hypercalcemia, starting with primary hyperparathyroidism. Intact PTH was found to be suppressed and neck ultrasound was unremarkable, making this diagnosis unlikely. Vitamin D testing demonstrated elevated calcitriol levels, which is seen in the clinical context of lymphoma and chronic granulomatosis disease [1, 2, 7]. These conditions were unlikely in our patient given lack of constitutional and inflammatory systemic symptoms, unremarkable bone scan, and chest CT and X-ray follow up ruled out the possibility of primary lung malignancy and active granulomatous diseases.

While hypercalcemia is seen in 10–20% of patients with Sarcoidosis, [3, 7] the diagnosis is unlikely in the absence of any physical exam, laboratory, or radiologic evidence of Sarcoidosis [2, 7]. Furthermore, low ACE levels make this diagnosis even less likely seeing that ACE levels are elevated in 60% of patients with Sarcoidosis [3]. Active tuberculosis infection is also associated with hypercalcemia [2] and was explored given our patient’s history of tuberculosis, but workup was negative. The patient denied taking vitamin D or A, silicon, or paraffin oil, ruling out external causes of hypercalcemia. One consideration is that while this patient underwent a broad workup, an underlying pathology explaining the observed hypercalcemia may reveal itself with time. This is a possibility so the authors will remain vigilant moving forward.

The first report discussing idiopathic calcitriol induced hypercalcemia emerged in 1994, where Kreisberg [1] explored all the possible etiologies of hypercalcemia and appropriate workups through clinical problem-solving. He never found a definitive diagnosis, and his patient improved on low dose prednisone that was continued long-term. Evron et al. [6] subsequently coined the term idiopathic calcitriol induced hypercalcemia suggesting it as a possible diagnosis for three patients they treated. Their patients presented with low iPTH, high calcitriol and high ACE levels. After finding no evidence of Sarcoidosis and normalization of calcium levels post prednisone treatment, they suggested term idiopathic calcitriol induced hypercalcemia as a separate disease entity. Rijckborst et al. [7] consolidated the diagnosis further describing one patient with low iPTH, high calcitriol and high ACE levels. Their patient also underwent a robust investigative workup demonstrating no clear etiology that could explain the observed hypercalcemia. Their patient also responded to prednisone but required a low maintenance dose to prevent disease recurrence. Our patient’s presentation and treatment course differ from those previously reported in respect to ACE levels. Elevated ACE levels in previous case reports [6, 7] lead them to postulate alternation in macrophage function as a possible underlying mechanism for idiopathic calcitriol induced hypercalcemia. Low ACE levels in our patient might emphasize the need to look for alternative causes that could explain elevated calcitriol and calcium levels. Additionally, our case report outlines a case of specific recurrent nephrolithiasis from a young age. Although idiopathic calcitriol induced hypercalcemia is not generally associated with nephrolithiasis, previous history of kidney stone can explain the susceptibility of nephrolithiasis in the setting of hypercalcemia and hypercalciuria.

Calcitriol plays a key role in calcium homeostasis. Systemically, calcitriol binds to vitamin-D receptors in the kidneys, parathyroid glands, intestines, and bones to raise serum blood calcium levels by facilitating intestinal absorption, renal tubular reabsorption, and bone release. The calcium-binding protein encoded by the transcription factor calcitriol carries both calcium and phosphate ions concurrently through intestinal epithelial cells [8]. By stimulating osteoclasts via the release of receptor activator of nuclear factor kappa-B ligand (RANKL) from osteoblasts, calcitriol promotes bone resorption. By causing apoptosis, calcitriol dramatically reduces the growth of T lymphocytes and normal human epidermal keratinocytes. Through this distinct array of self-regulatory mechanisms, calcitriol increases serum calcium levels. Therefore, elevated calcitriol levels idiopathically will lead to hypercalcemia that is resistant to feedback pathways that usually reduce parathyroid hormone production and decreases calcium levels. Corticosteroids inhibit the production of calcitriol in macrophages, which reduces gut absorption of calcium, RANKL expression, and would theoretically promote bone retention reducing calcium levels overall [9]. However, in reality, long term use of glucocorticoids can result in a variety of side effects including bone weakening. Glucocorticoid can activate osteoclast first, later can inhibit osteoblast, which can cause osteoporosis, fractures and osteonecrosis [10]. Long term medium or high dose glucocorticoid use can cause cushingoid facial features, buffalo hump, induce refractory hyperglycemia or worsen existing diabetes, increase risk of infection, and cause a variety of other harmful effects [11]. Titrating the steroid dosage to ensure symptoms do not return, while minimizing the overall steroid burden can be difficult. Yet, this down titration process is necessary to ensure appropriate treatment of Idiopathic Calcitriol Induced Hypercalcemia while minimizing unintended consequences. While the patient continues steroid therapy, it is necessary to monitor for long-term toxicity associated with this treatment. For instance, bone mineral density (BMD) will need to be monitored one year after steroid initiation with appropriate follow-up screening every two years if BMD is stable [10]. In addition to BMD, other parameters that require surveillance with long-term steroid use include lipids, glucose parameters, and intraocular pressures. Authors are considering alternative management in the future. One such alternative is to trial Mycophenolate mofetil, an immunosuppressive agent that has been effective in the treatment of Sarcoidosis [12].

In summary, this case report discusses the case of a 51-year-old male with symptomatic hypercalcemia in the setting of Idiopathic Calcitriol Induced Hypercalcemia. Treatment with prednisone showed marked improvement of symptoms and long-term management continues to be optimized. This report builds on previous literature unifying evidence supporting Idiopathic Calcitriol Induced Hypercalcemia as a distinct disease entity while simultaneously reinforcing the need to explore alternative hypotheses for disease pathogenesis.

While identifying information was not included in this report, written informed consent was obtained from the patient to share this report in an online open-access publication.

## Electronic supplementary material

Below is the link to the electronic supplementary material.

Supplementary Material 1

## Data Availability

The patient data used during this report is available from the corresponding author on reasonable request and was submitted to BMC Nephrology.

## List of abbreviations

Calcitriol: 1,25(OH)_2_D
iPTH: hypercalcemia included intact PTH
calcidiol: 25-hydroxyvitamin D
PTH: parathyroid hormone
PTHrP: parathyroid hormone-related peptide
RANKL: receptor activator of nuclear factor kappa-B ligand
